# Supplementary material for: Polyketide synthase gene clusters in the ericoid mycorrhizal fungus Oidiodendron maius: genome-wide prediction, expression profiling, and functional characterization of knockout mutants
Source: Front Plant Sci. 2026 Sep 16;17:1924430. doi: 10.3389/fpls.2026.1924430 (PMC13623903; doi:10.3389/fpls.2026.1924430)
Supplement: Supplementary file 1 [file SupplementaryFile1.zip › Supplementary Materials/Supplementary Folder 1.zip!/Oidma1_AssemblyScaffolds_Repeatmasked/knownclusterblast/cluster49/jgi.p_Oidma1_136491_mibig_hits.html]

| MIBiG Protein | Description | MIBiG Cluster | MiBiG Product | % ID | % Coverage | BLAST Score | E-value |
| --- | --- | --- | --- | --- | --- | --- | --- |
| BAQ25463.1 | short-chain\_dehydrogenase | BGC0001280 | Betaenone\_C\_/\_betaenone\_A\_biosynthetic\_gene\_cluster | 39 | 101.230769231 | 237 | 2.3e-62 |
| CDM31314.1 | Short-chain\_dehydrogenase/reductase\_SDR | BGC0000667 | PR\_toxin\_biosynthetic\_gene\_cluster | 32 | 68.0 | 119 | 9.2e-27 |
| AAF81727.1 | putative\_ketoreductase\_EncD | BGC0000220 | Enterocin\_biosynthetic\_gene\_cluster | 27 | 82.1538461538 | 50 | 4e-06 |
